# Do left-wingers discriminate? A cross-country study on the links between political orientation, values, moral foundations, and the Covid-19 passport

**DOI:** 10.1007/s12144-023-04554-9

**Published:** 2023-03-21

**Authors:** Gabriel Lins de Holanda Coelho, Lukas J. Wolf, Roosevelt Vilar, Renan Pereira Monteiro, Paul H. P. Hanel

**Affiliations:** 1 University College Cork, Cork, Ireland; 2 University of Bath, Bath, UK; 3 Universitas Sebelas Maret, Surakarta, Indonesia; 4 Federal University of Paraíba, João Pessoa, Brazil; 5 University of Essex, Colchester, UK

**Keywords:** Covid-19 passport, Vaccine, Attitudes, Discrimination, Human values, Moral Foundations

## Abstract

To increase Covid-19 vaccine uptake and protect vulnerable people, many countries have introduced a Covid-19 passport in 2021, allowing vaccinated individuals to access indoor facilities more freely and travel to foreign countries. However, the passport has had unintended consequences as it discriminates against those who do not want to get vaccinated for medical, religious, or political reasons, or those who do not have access to vaccines. The present study (*N* = 678) assessed across Brazil, UK, USA, and a group of other countries, the links between political orientation, human values, and moral foundations, and attitudes towards the Covid-19 passport and whether people perceive it as a discriminatory measure. Results showed that left-wingers, typically more inclined to recognize discrimination, favor the passport more and perceive it as less discriminatory than right-wingers. This pattern remains consistent even after controlling for human values and moral foundations, independently predicting attitudes towards the passport. Overall, our findings provide novel insights into a context in which left-wingers support measures that involuntarily discriminate against certain groups.

**Supplementary information:**

The online version contains supplementary material available at 10.1007/s12144-023-04554-9.

## Introduction

The Covid-19 pandemic has provided humanity with unprecedented challenges. Many debates have erupted about the best measures to contain the spread of the virus while ideally keeping schools and other venues open and protecting vulnerable people (Giubilini et al., 2020). One measure that led to heated and polarizing debates is the Covid-19 passport (also known as “Covid-19 Certificate” or “Health Passport”). This digital certificate provides access to venues and international travel for those with proof of vaccination, a negative test, or recovery (European Commission, 2021). It has been argued that the Covid-19 passport increases vaccine uptake and offers safety and reassurance in indoor social spaces such as restaurants and cinemas (Sharif et al., 2021; Sotis et al., 2021). The use of Covid-19 certificates has been celebrated by sectors that severely suffered the consequences of the pandemic, such as hospitality or airlines, that consider it a tool that allows resuming travel and tourism (Quinn, 2021).

However, implementing the Covid-19 passport has also opened a debate on whether the measure is appropriate. One of the most common arguments is that it implies discrimination against specific groups, such as those who do not want to get vaccinated for medical, religious, or political reasons (Schraer, 2021). Additionally, in many countries, vaccines were rolled out among older citizens first, meaning that young people have had to wait longer to return to their daily social activities, even if they intend to get vaccinated. Also, people who belong to minority groups, such as Black Caribbean and Black African people in the UK, have substantially lower vaccine uptake (Office for National Statistics, 2022), often because they tend to trust governments less (Razai et al., 2021). Interestingly, minority members are even less in favor of vaccinating than conservatives (Latkin et al., 2021a). However, the largest group of people impacted by the lack of vaccines are those living in low-income countries. According to the Global Dashboard for Vaccine Equity (Data Futures Platform, 2022), as of March 30, 2022, only 15.23% of the population in low-income countries have been vaccinated with at least one dose, compared to 71.75% in high-income countries. This issue comes from ethical concerns about the globally unequal distribution of vaccines (Rzymski et al., 2021).

Therefore, given the polarizing nature of the perceived benefits and risks of implementing a Covid-19 passport, it is crucial to gain more insight into what can explain people’s diverging views. This is particularly important even if the passport is no longer compulsory in many countries (BBC News, 2022) since, in the future, policymakers might consider re-introducing the passport if new viruses emerge, for instance. In the present research, we assess across multiple countries whether political orientation, human values, and moral foundations play a significant role in explaining peoples’ attitudes towards the Covid-19 passport and whether they perceive it as discriminatory towards those who chose not to get vaccinated.

### Association of discrimination with political orientation, human values, and moral foundations

Prior work has identified a range of constructs that underpin discrimination against those who are often disadvantaged in various ways. For instance, on the political spectrum, right-wing authoritarianism strongly predicts prejudice against underrepresented racial groups and homosexuals (Laythe et al., 2001). In contrast, a left-wing orientation positively predicts support for legal protection against discrimination based on sexual orientation (Yeo & Chu, 2018).

Such links with discrimination align with the human values frequently endorsed by those political groups. Human values represent desirable goals that differ in importance (Schwartz, 1992). In Schwartz’s theory of basic human values, values (e.g., benevolence, conformity) are organized under four higher-order values types, which are ordered along two dimensions (Coelho et al., 2019): openness (e.g., freedom, an exciting life) vs. conservation (e.g., security, tradition), and self-transcendence (e.g., equality, loyalty) vs. self-enhancement (e.g., success, wealth). Previous research found that self-transcendence values are frequently associated with left-leaning and more favorable views toward underrepresented groups, whereas conservation values are associated with a right-leaning political orientation and less favorable views toward these groups (Caprara et al., 2006; Davidov et al., 2008; Wolf et al., 2019).

Moral foundations are other essential variables which can be used to understand why some people hold negative views towards certain groups. The Moral Foundations Theory (Haidt & Joseph, 2004), distinguishes between five morality foundations that intend to represent our “intuitive ethics”, or innate ability to approve or disapprove of different behaviors. These five foundations are (Haidt & Joseph, 2004): Care, fairness, loyalty, authority, and sanctity. While values and moral foundations are correlated, they are conceptually independent constructs: Values are abstract ideals that guide people’s behavior while moral foundations explain differences in moral judgment (Feldman, 2021; Zapko-Willmes et al., 2021). The differences between the two sets of constructs become further apparent when we look at the measures themselves: Value measures (e.g., Schwartz, 1992) ask about the importance of abstract ideals such as equality or success, whereas moral foundation questionnaires (e.g., Graham et al., 2011) ask about whether specific feelings or actions of people are right or wrong. Notably, past evidence suggests that individuals with higher loyalty, authority, and sanctity show higher support for discrimination against refugees or Muslims. In contrast, people with higher levels of care and fairness showed less prejudicial attitudes (Captari et al., 2019; Kugler et al., 2014).

At first glance, these previous findings suggest that being left-leaning, endorsing self-transcendence values, and having high levels of care and fairness moral foundations describe someone unlikely to discriminate against social groups. However, this pattern might shift depending on the targeted groups and whether these conflict with the left-wingers’ progressive agenda. For example, a recent study showed that liberals (left-wingers) blamed the unvaccinated for the continuation of the pandemic more than the vaccinated (Graso et al., 2022). In contrast, conservatives were unlikely to scapegoat either group. This suggests that left-wingers are more likely to be vaccinated people who feel antipathy towards the unvaccinated ones (Bor et al., 2022), potentially because the unvaccinated outgroup is perceived to have more control over their group membership, while they are more protective of those who cannot choose their group membership freely. In line with this notion, left-wingers have been found to discriminate against outgroups such as right-wingers (Frimer et al., 2017; Hanel et al., 2018) and Christians (Badaan & Jost, 2020). This might be because right-wingers and Christians show signs of intolerance toward groups such as homosexuals (e.g., Barton, 2010; Laythe et al., 2001), which are seen favorably by left-wingers.

In countries where vaccines are available, getting vaccinated and using a Covid-19 passport might be perceived as under most people’s control. Further, left-wingers might perceive unvaccinated people as selfish because getting vaccinated has been framed as an act of solidarity and social harmony. Hence, it might be expected that those with more left-leaning beliefs are less likely to be concerned about any potential discrimination or unfairness that those passports may bring for the unvaccinated. In contrast, right-wingers are less likely to trust science (Agley, 2020), which may be essential in rejecting vaccines and Covid-19 passports. Indeed, research has shown that right-wingers report firmer free-will beliefs and value individual responsibility more (Chan, 2019) than left-wingers (Everett et al., 2021; see also Nowlan & Zane, 2020). Because right-wingers may believe that getting vaccinated is a personal choice, they may be less likely to discriminate against the unvaccinated.

### The present research

Previous studies established that left-wingers, people who strongly endorse self-transcendence values, and those higher on the care and fairness moral foundations feel more positively towards groups typically disadvantaged than right-wingers and people scoring lower on all these variables. In the present research, we test whether this pattern is reversed when considering the Covid-19 passport. Ideologically, left-wingers tend to hold more positive attitudes toward the vaccine and vaccination policies than right-wingers (Kossowska et al., 2021), showing care for those in more vulnerable situations (e.g., the elderly, people with comorbidities). However, this support might result in a “side-effect”. Tools such as the Covid-19 passport can involuntarily negatively affect groups typically backed by left-wingers, such as underrepresented groups (Office for National Statistics, 2022) and people from low-income countries (Ritchie et al., 2020), since they are less likely to be vaccinated (Latkin et al., 2021a). In other words, this discrimination would presumably be involuntary, as it is not directed at these affected groups but at those who freely chose not to get the vaccine. On the other hand, in most developed countries, getting vaccinated is possible and recommended by scientists, who are trusted more by left-wingers (Agley, 2020). Further, getting vaccinated is vital as it helps reduce the overall number of hospitalizations and protects those more vulnerable such as the elderly and people with weak immune systems (Giubilini et al., 2020).

Based on the evidence reviewed above, we therefore expect left-wingers to be more in support of the Covid-19 passport and perceive it as a less discriminatory tool than right-wingers. Further, we expect values and moral foundations typically associated with left-wingers, such as universalism, care, and fairness (Caprara et al., 2006; Graham et al., 2011), to be positively associated with support for the Covid-19 passport. Conversely, we expect values and moral foundations typically associated with right-wingers, such as conformity, tradition, security, authority, loyalty, and sanctity, to be negatively associated with support for the Covid-19 passport. Together, our study will provide new insights into the political and psychological impact on the perception of discrimination when the discriminated target appears free to choose their group membership (e.g., non-vaccinated).

More specifically, we will assess whether human values and moral foundations predict people’s attitudes towards Covid-19 passports and whether they perceive passports as discriminatory, above and beyond political orientation, while also controlling for participants’ country of origin. In our study, we used samples from the United States, Brazil, the United Kingdom, and a group of other countries (e.g., Germany, Ireland, Canada). These countries were among the most severely impacted by the virus (Worldometer, 2022), and used different approaches to deal with the pandemic. However, we have not made any specific predictions whether country moderates any effect. Finally, Data and supplementary table (e.g., participants’ demographics) are available as Online Supplementary Material (OSM: https://osf.io/jpwum/?view_only=8a99dc69c9624ee49ef92d9ce2ce790b).

## Method

### Procedure and participants

A power calculation revealed that a sample size of 139 was required to detect an effect size of *r* = .30 with a 95% power in each country. We aimed for that sample size per country to test whether the proposed effects replicate across countries.

We created and advertised English and Brazilian-Portuguese versions of the survey on social media (e.g., Facebook, Reddit, Instagram). The only inclusion criteria for participation in our study were 18 + years old and fluent in English or Portuguese. Also, because we did not reach an adequate number of participants in some countries (e.g., Canada, Germany, Ireland), we combined them into a single group (i.e., “Other countries”). Our final sample consisted of 678 individuals (*M*_*age*_ = 32.21; *SD*_*age*_ = 12.38) residing in different countries (i.e., United States = 199 or 29.4%; Brazil = 233 or 34.4%; United Kingdom = 106 or 15.6%; Other countries = 140 or 20.6%). Participants identified their gender as woman (*n* = 379 or 55.9%), man (*n* = 262 or 38.6%), or non-binary (*n* = 26 or 3.8%). They described their political orientation as left-wing (*n* = 216 or 31.9%), centre-left (*n* = 136 or 20.1%), or right-wing (*n* = 76 or 11.3%). Ninety-nine (14.6%) had been positively tested for Covid-19, and 364 (53.7%) were fully vaccinated. Participants who reported not knowing what the Covid-19 Passport is, were informed.

To identify careless responses, we added “test items” throughout the questionnaires to check attention, i.e., asking to select a specific answer category in one of the questionnaires (e.g., “Please select *Strongly Disagree*”). These participants were dropped from the dataset before the analyses and were not counted toward the final sample. Participants who finished the study at a questionably fast pace were also removed. In total, 30 participants were excluded. Supplemental Table S1 shows the demographics per country (OSM).

### Material

Participants completed the survey in English or Brazilian-Portuguese. We used existing validated versions of scales or translated them using the back-translation of experienced bilingual researchers. We used the following questionnaires:

To assess human values, we used an adapted version of the *Portrait Value Questionnaire* (PVQ; Schwartz, 2003), composed of 21 items covering the ten value types (e.g., benevolence, power, hedonism) from Schwartz’s model. We asked participants to rate how much each statement describes themselves instead of an abstract female or male person, using a six-point scale (1 = *Very much like me*; 6 = *Not like me at all*). Items included “*Tradition is important to me. I try to follow the customs handed down by my religion or my family*” (Tradition) and “*It is important to me to live in secure surroundings. I avoid anything that might endanger my safety*” (Security). In Brazil, we used the version translated by Tamayo and Porto (2009). In our study, the inter-item correlations ranged from 0.16 (Tradition) to 0.56 (Hedonism) for the value types^1^. To further test the reliability of the PVQ-21, we performed a multidimensional scaling analysis (Bilsky et al., 2011), which groups the ten value types based on their associations with each other in a two-dimensional space. In other words, value types that are more strongly associated with each other, are placed next to each other. Our data replicates Schwartz’s (1992) model well (see Online Supplemental Materials), thus providing further evidence for the reliability of our data.

Furthermore, we used a 20-item version of the Moral Foundations Questionnaire (MFQ; Graham et al., 2011), available on the authors’ website (https://moralfoundations.org/questionnaires/). Four items measure each of the five moral foundations: Care, fairness, loyalty, authority, and sanctity. The questions are divided into two sets, that use different response scales. In the first set, participants indicate to what extent the items are relevant to their thinking (e.g., *Whether or not some people were treated differently than others*), using a six-point scale (1 = *Not at all relevant [This consideration has nothing to do with my judgments of right and wrong]*; 6 - *Extremely relevant [This is one of the most important factors when I judge right and wrong]*). In the second set, they indicate their level of agreement with each item (e.g., *Compassion for those who are suffering is the most crucial virtue*.), using a six-point scale (1 = *Strongly Disagree;* 6 = *Strongly Agree*). The questionnaire was validated in Brazil by Moreira et al. (2019). In our study, the reliabilities (McDonald’s omega, ω; Cronbach’s alpha, α) of the individual moral foundations ranged from 0.63 (fairness) to 0.74 (sanctity) for McDonald’s omega, and from 0.63 (fairness) to 0.73 (sanctity) for Cronbach’s alpha.

Moreover, we created a questionnaire to assess attitudes towards the Covid-19 passport (ω and α = 0.98). Participants were asked to evaluate how they perceived the Covid-19 passport. Following the format to measure attitudes used by Armitage et al. (1999), we created eight bipolar items whereby the scale end-points varied from − 3 to 3 on a 7-point scale (*bad-good, useless-useful, unfavorable-favorable, negative-positive, difficult-easy, unfair-fair, unnecessary-necessary, unreliable-trustworthy*).

To measure whether participants perceive the passport as discriminatory, we asked them to indicate the extent to which they think the Covid-19 passport (ω = 0.86; α = 0.80): (1) … *can divide society between those with it, and those without such passport?;* (2) … *is discriminatory*?; (3) … *is fair, as those unvaccinated will not be allowed in some public places?* (reversed); and (4) … *could harm social cohesion in your country*? Answers were given on a 101-point scale from 0% (*Not at all*), to 100% (*Totally*).

Finally, we assessed political orientation with a single item. Responses were given on a 7-point scale ranging from extreme left (1) to extreme right (7). Higher scores hence reflect a more right-wing orientation.

### Data analysis

Data were analyzed using the open-source software JAMOVI (https://www.jamovi.org/) and JASP (https://jasp-stats.org/). We performed an analysis of variance (ANOVA) to test for cross-cultural mean differences, Pearson’s correlation, and multiple linear regressions.

## Results

**Fig. 1:**
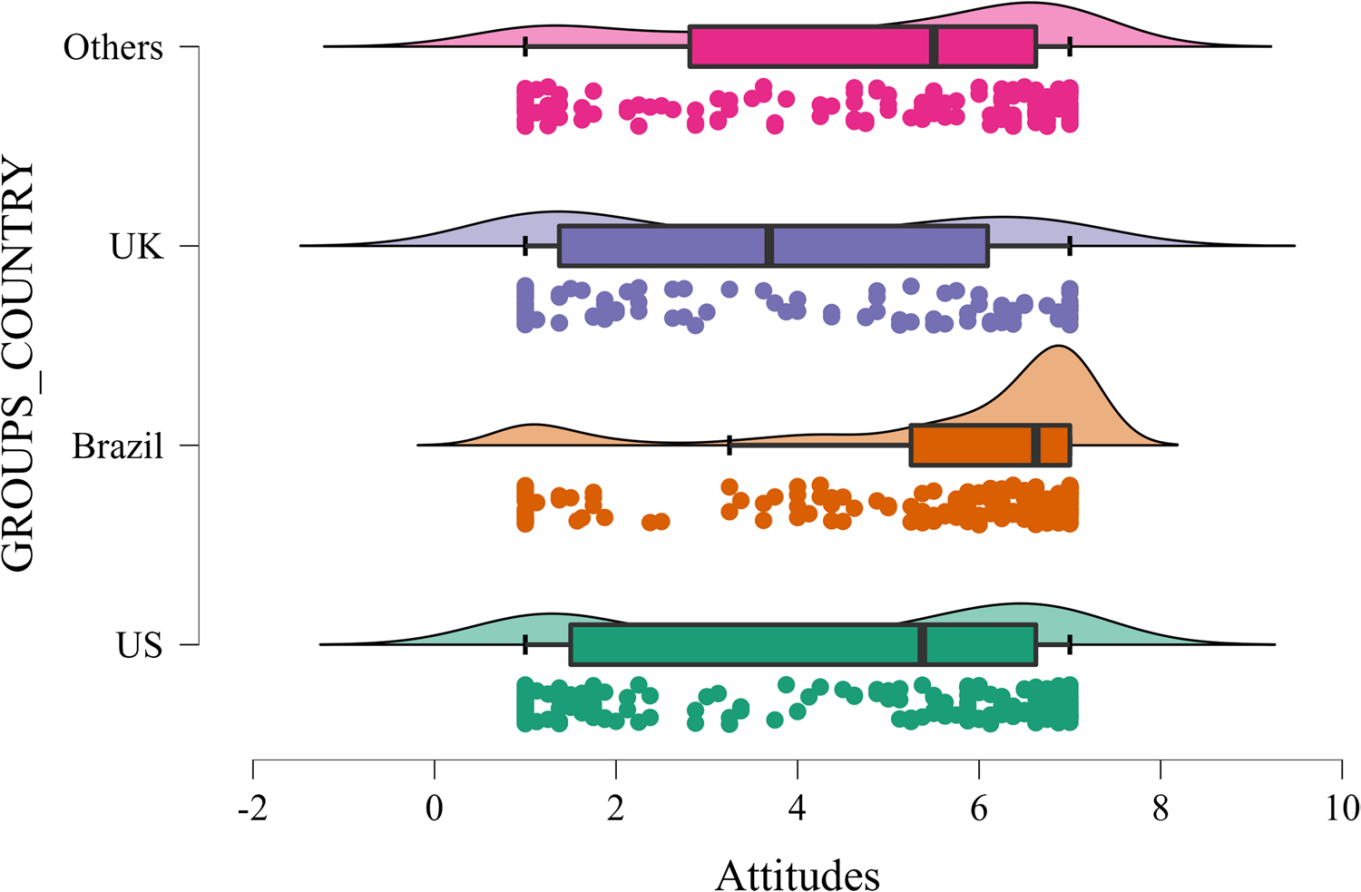
Attitudes towards the Covid-19 passport across countries

**Fig. 2:**
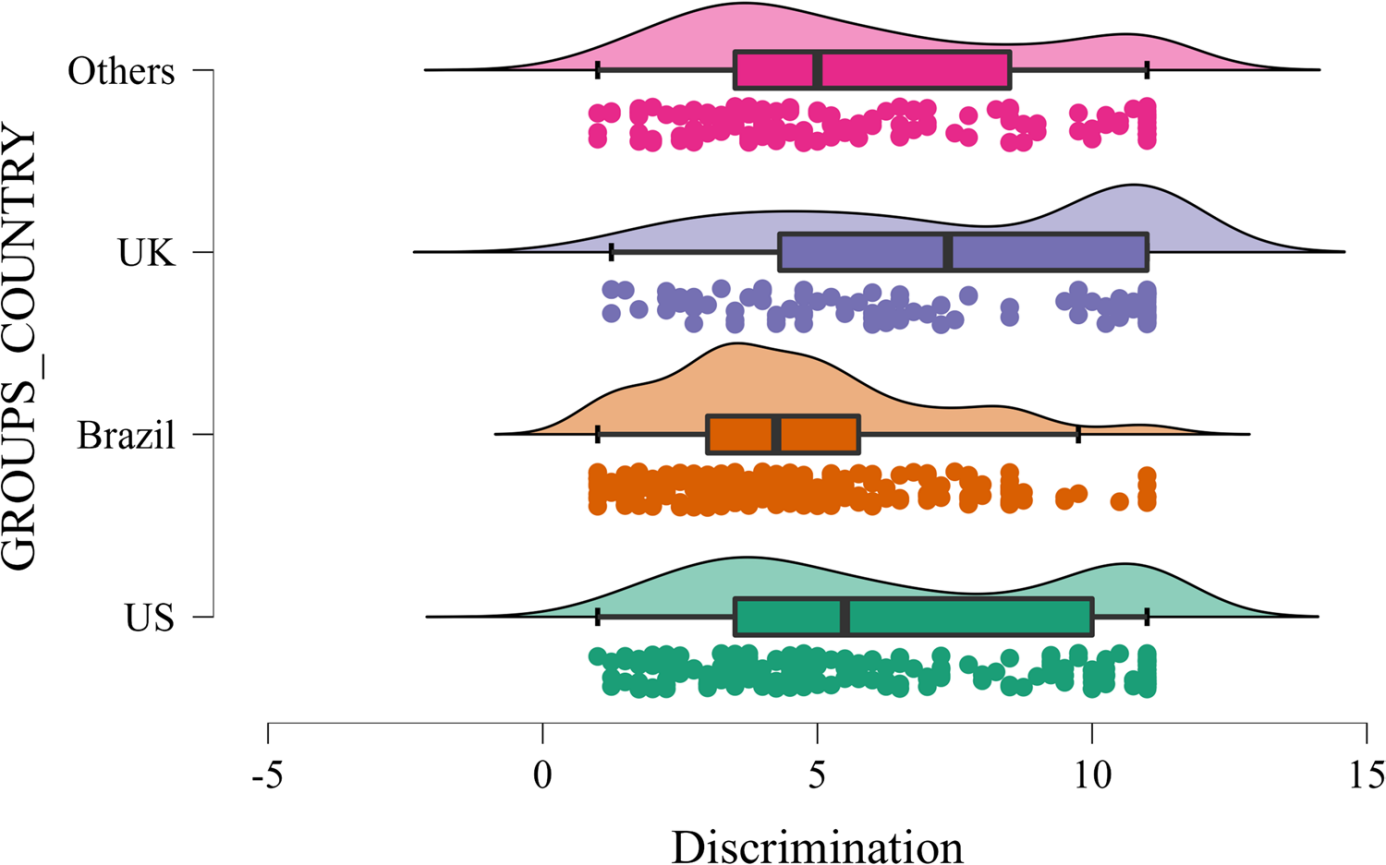
COVID-19 perceived discrimination across countries

First, we conducted two one-way between-subject ANOVAs test mean differences between people living in the United States (*M*_att_ = 4.31, *SD*_att_ = 2.41; *M*_disc_ = 6.41, *SD*_disc_ = 3.23), Brazil (*M*_att_ = 5.60, *SD*_att_ = 2; *M*_disc_ = 4.57, *SD*_disc_ = 2.37), United Kingdom (*M*_att_ = 3.75, *SD*_att_ = 2.33; *M*_disc_ = 7.37, *SD*_disc_ = 3.38), and “other” countries (e.g., Germany, Ireland ; *M*_att_ = 4.68, *SD*_*a*tt_ = 2.21; *M*_disc_ = 5.77, *SD*_disc_ = 3.12) on attitudes towards the Covid-19 passport, *F*(3, 673) = 21.200, *η*^2^ = 0.086, *p* < .001, and perceived discrimination, *F*(3, 673) = 25.577, *η*^2^ = 0.102, *p* < .001. Posthoc tests using Sidak correction showed that people living in Brazil reported more positive attitudes towards the passport than people from all other countries (*ps* < 0.001). Further, people in the UK also reported more negative attitudes than those in “Other countries”, *p* = .007 (Fig. 1). People living in the UK perceived the passport on average as more discriminatory than people in the US, *p* = .046, Brazil, *p* < .001, and “Other countries”, *p* < .001, whereas participants living in Brazil perceived the passport less discriminatory than people from the three other groups, *ps* ≤ 0.001 (Fig. 2).

As seen in Table 1, attitudes towards the passport and perceived discrimination were strongly negatively correlated, except in Brazil, where correlations were still significantly negative, *r* = − .34 (*p* < .001) but less strong than in the other three groups, *r*s = − 0.78 (*p* < .001) to − 0.86 (*p* < .001). Despite the strong negative correlations, we decided to keep attitudes and perceived discrimination for all further analyses separate, because perceived discrimination is a main focus of the present study. Conceptually, while attitudes represent the overall evaluation of the passport, perceived discrimination is a specific belief about Covid-19 passports that may be assumed to feed into the overall attitude. We, therefore, treat overall attitude and perceived discrimination as closely linked but distinguishable.

**Table 1:** Correlation matrix

	US				BR				UK			Others			
	1		2		1			2	1		2	1			2
1. Attitudes	-				-				-			-			
2. Pass Discrimination	− 0.852**				− 0.337**				− 0.858**			− 0.778**			
Political Orientation	− 0.711**		0.702**		− 0.269**			0.430**	− 0.573**		0.582**	− 0.595**			0.560**
Values															
Security	0.202**		− 0.230**		0.243**			− 0.169*	0.260*		− 0.190	0.369**			− 0.253**
Conformity	0.066		− 0.049		0.078			0.056	0.300**		− 0.241*	0.193*			− 0.207*
Tradition	− 0.356**		0.397**		− 0.070			0.190**	− 0.227**		0.354**	− 0.182*			0.233**
Benevolence	0.108		− 0.095		0.018			− 0.073	− 0.012		0.032	0.030			0.011
Universalism	0.295**		− 0.280**		0.108			− 0.201**	0.148		− 0.127	0.255**			− 0.192*
Self Direction	− 0.110		0.122		− 0.060			− 0.006	− 0.278**		0.189	− 0.191*			0.151
Stimulation	− 0.116		0.078		− 0.016			− 0.046	0.090		− 0.112	− 0.056			− 0.039
Hedonism	0.085		− 0.155*		0.087			− 0.047	0.281**		− 0.284**	0.137			− 0.126
Achievement	0.000		− 0.022		0.017			− 0.067	0.323**		− 0.287**	0.063			− 0.223**
Power	0.005		0.016		0.010			− 0.028	0.279**		− 0.246*	0.093			− 0.142
Moral Foundations															
Care	0.377**		− 0.372**		0.300**			− 0.114	0.248*		− 0.169	0.302**			− 0.319**
Fairness	0.238**		− 0.240**		0.265**			− 0.185**	0.134		− 0.023	0.095			− 0.137
Loyalty	− 0.550**		0.552**		− 0.179**			0.317**	− 0.312**		0.371**	− 0.358**			0.292**
Authority	− 0.530**		0.554**		− 0.107			0.230**	− 0.277**		0.377**	− 0.299**			0.280**
Sanctity	− 0.368**		0.378**		− 0.085			0.225**	− 0.308**		0.357**	− 0.171*			0.221**

In bold = Significant correlations; * *p* < .05; ** *p* < .01; Higher numerical values represent higher endorsement. For example, higher scores on “Attitudes” indicate more favourable views of the Covid-19 passport

Further, we found that political orientation was negatively correlated with attitudes and positively with perceived discrimination in all countries. Nevertheless, the country moderated how political orientation is associated with attitudes and perceived discrimination. Moderated regression analyses confirmed that the relation was weaker in Brazil compared to the UK, the US, and other countries (see OSM).

Moreover, we found that perceived discrimination and attitudes towards the Covid-19 passport correlate with political orientation, human values, and moral foundations across all countries (Table 1). As predicted, individuals had more positive attitudes towards the passport and perceived the passport as less discriminatory when they were left-wingers, valued universalism more and conservative values such as security and tradition less, and scored higher on care and fairness, but lower on loyalty, authority, and sanctity foundations.

Next, we performed two multiple linear hierarchical regressions with political orientation, human values, and moral foundations as predictors of attitudes toward the Covid-19 passport and its perceived discrimination. We also included countries as dummy-coded control variables in these models, with Brazil as a reference country because people there scored differently from the other three groups (Table 2). Political orientation and country were included in step one (Model one), and human values and moral foundations were included in step two (Model two). In other words, Model one considered only political orientation and country, whereas Model two considered all variables. Political orientation, three value types (security, conformity, self-direction), and two moral foundations (care and loyalty) predicted both dependent variables independently. In contrast, benevolence only predicted attitudes, and achievement only predicted perceived discrimination. This suggests that left-wingers keep perceiving the passport as more positive and less discriminatory even after controlling for various values and moral foundations. The inclusion of values and moral foundations in step two significantly increased the variance explained by the model (attitudes, Δ*R²* = 0.08, *p* < .001; perceived discrimination, Δ*R²* = 0.07, *p* < .001) beyond the contribution of political orientation and country of origin.

**Table 2:** Results of multiple regressions

	Attitudes			Discrimination	
	Model 1		Model2	Model 1	Model 2
* F(df)*	92.040 (4, 652)		26.700 (19, 637)	115.097 (4, 652)	31.322 (19, 637)
*R²*	**0.36 (< 0.001)**		**0.44(< 0.001)**	**0.41(< 0.001)**	**0.48(< 0.001)**
Δ *R²*	**0.08 (< 0.001)**			**0.07 (< 0.001)**	
	**β** ***(p)***			**β** ***(p)***	
Political Orientation	**-0.537 (< 0.001)**		**− 0.353 (< 0.001)**	**0.568 (< 0.001)**	**0.381 (< 0.001)**
Countries:					
US – Brazil	**-0.453 (< 0.001)**		**− 0.345 (< 0.001)**	**0.493 (< 0.001)**	**0.439 (< 0.001)**
UK – Brazil	**-0.543 (< 0.001)**		**− 0.439 (< 0.001)**	**0.637 (< 0.001)**	**0.567 (< 0.001)**
Others - Brazil	**-0.441 (< 0.001)**		**− 0.357 (< 0.001)**	**0.459 (< 0.001)**	**0.434 (< 0.001)**
Values					
Security			**0.148 (< 0.001)**		**− 0.131 (< 0.001)**
Conformity			**0.073 (0.05)**		**− 0.077 (0.032)**
Tradition			− 0.005 (0.888)		0.07 (0.06)
Benevolence			**− 0.08 (0.028)**		0.059 (0.09)
Universalism			− 0.039 (0.337)		0.039 (0.314)
Self Direction			**− 0.071 (0.036)**		**0.068 (0.037)**
Stimulation			− 0.006 (0.864)		− 0.045 (0.212)
Hedonism			0.032 (0.382)		− 0.012 (0.734)
Achievement			0.04 (0.29)		**− 0.084 (0.02)**
Power			0.026 (0.496)		0.022 (0.552)
Moral Foundations					
Care			.**18 (< 0.001)**		**− 0.123 (0.003)**
Fairness			0.005 (0.902)		− 0.007 (0.862)
Loyalty			**− 0.109 (0.014)**		**0.097 (0.023)**
Authority			− 0.074 (0.131)		0.075 (0.113)
Sanctity			− 0.049 (0.242)		0.063 (0.119)

Bold = significant

Finally, we explored whether those associations differed across countries. The results can be seen in Table 3. Political orientation was a significant predictor in seven of the eight regressions (4 countries x 2 dependent variables): Right-wingers generally had more negative attitudes towards the passport and perceived the passport as more discriminatory across countries. The results for values and moral foundations were similar to the findings for the whole sample, but the findings suggest some differences between countries. For instance, security (in the US and “other countries”) and conformity values (in the UK) as well as the care foundation (in Brazil), positively predicted attitudes towards the passport. Also, security (in the US and “other countries”) and conformity values (in the UK), as well as fairness (in Brazil) and care foundations (“Other countries”) were negatively associated with perceived discrimination. Curiously and differently from the general results, some other values and moral foundations were significant predictors of attitudes or perceived discrimination in some groups. For instance, authority foundations predicted attitudes negatively and perceived discrimination positively in the US, and achievement values positively predicted attitudes in the UK. Despite these differences, the general pattern suggests that conservation values (i.e., security and conformity values) and individualizing moral foundations (i.e., care and fairness) predict more positive attitudes and lower perceived discrimination.

**Table 3:** Results of multiple regressions per country

	US		BR		UK		Others	
	Attitudes	Discrimination	Attitudes	Discrimination	Attitudes	Discrimination	Attitudes	Discrimination
*F(df)*	14.995 (16, 180)	17.171 (16, 181)	3.275 (16, 201)	5.234 (16, 200)	6.363 (16, 88)	5.455 (16, 88)	7.660 (16, 120)	7.278 (16, 120)
*R*^*2*^	0.57	0.60	0.21	0.29	0.54	0.50	0.50	0.49
	**β** ***(p)***		**β** ***(p)***		**β** ***(p)***		**β** ***(p)***	
Political Orientation	**− 0.514 (< 0.001)**	**0.452 (< 0.001)**	− 0.114 (0.146)	**0.313 (< 0.001)**	**− 0.504 (< 0.001)**	**0.435 (< 0.001)**	**− 0.389 (< 0.001)**	**0.343 (< 0.001)**
Values								
Security	**0.145 (0.022)**	**− 0.206 (< 0.001)**	0.159 (0.051)	− 0.116 (0.13)	0.047 (0.617)	− 0.071 (0.473)	**0.315 (< 0.001)**	**− 0.168 (0.044)**
Conformity	0.061 (0.366)	− 0.060 (0.350)	0.033 (0.68)	0.045 (0.545)	**0.205 (0.033)**	**− 0.256 (0.011)**	0 (0.996)	− 0.131 (0.124)
Tradition	− 0.022 (0.741)	0.051 (0.428)	0.009 (0.908)	0.087 (0.262)	0.032 (0.75)	0.141 (0.173)	0.014 (0.864)	0.005 (0.956)
Benevolence	− 0.064 (0.284)	0.091 (0.115)	− 0.083 (0.303)	− 0.026 (0.733)	− 0.06 (0.494)	0.053 (0.562)	− 0.103 (0.194)	0.154 (0.056)
Universalism	− 0.109 (0.152)	0.110 (0.134)	− 0.09 (0.283)	− 0.04 (0.615)	− 0.139 (0.198)	0.132 (0.238)	0.077 (0.36)	− 0.018 (0.829)
Self Direction	0.002 (0.979)	0.034 (0.530)	− 0.034 (0.633)	0.023 (0.734)	− 0.144 (0.085)	0.066 (0.444)	− 0.073 (0.386)	0.113 (0.186)
Stimulation	− 0.078 (0.256)	0.077 (0.242)	0.017 (0.818)	− 0.091 (0.203)	0.025 (0.806)	− 0.064 (0.542)	0.02 (0.819)	− 0.109 (0.224)
Hedonism	0.049 (0.478)	**− 0.149 (0.026)**	0.077 (0.289)	0.021 (0.76)	− 0.074 (0.431)	0.048 (0.621)	− 0.026 (0.729)	0.089 (0.246)
Achievement	0.013 (0.834)	− 0.052 (0.385)	0.03 (0.721)	− 0.046 (0.56)	**0.188 (0.042)**	− 0.15 (0.116)	0.008 (0.925)	**− 0.184 (0.026)**
Power	− 0.028 (0.668)	0.115 (0.065)	0.013 (0.875)	0.037 (0.638)	0.059 (0.529)	− 0.004 (0.968)	0.109 (0.162)	− 0.102 (0.194)
Moral Foundations								
Care	0.11 (0.122)	− 0.101 (0.131)	**0.251 (0.006)**	0.061 (0.483)	0.151 (0.171)	− 0.12 (0.295)	**0.215 (0.028)**	**− 0.297 (0.003)**
Fairness	0.018 (0.786)	− 0.014 (0.826)	0.079 (0.357)	**− 0.183 (0.024)**	0.071 (0.492)	0.056 (0.602)	− 0.139 (0.106)	0.093 (0.287)
Loyalty	− 0.069 (0.391)	0.029 (0.711)	− 0.171 (0.065)	**0.243 (0.006)**	0.041 (0.743)	− 0.051 (0.693)	− 0.05 (0.582)	− 0.013 (0.883)
Authority	**− 0.231 (0.017)**	**0.333 (< 0.001)**	− 0.018 (0.858)	− 0.09 (0.339)	0.037 (0.804)	0.095 (0.546)	− 0.148 (0.109)	0.126 (0.177)
Sanctity	0.041 (0.567)	− 0.060 (0.378)	− 0.064 (0.469)	0.036 (0.669)	**− 0.27 (0.017)**	0.151 (0.194)	− 0.047 (0.583)	0.144 (0.096)

Bold = significant

## Discussion

In the present research, we assessed whether political orientation, human values, and moral foundations significantly explain people’s attitudes toward the passport and whether they perceive it as discriminatory. We recruited samples from countries severely impacted by the virus: the United States, Brazil, and the United Kingdom, and a sample comprised of participants from a mixture of countries (e.g., Germany, Canada, Ireland).

### The role of political orientation, values, and moral foundations

We found that participants leaning towards the right end of the political spectrum had more negative attitudes towards the passport and perceived it as more discriminatory than left-wingers. Prior research suggests that some of the main reasons for left-wingers’ favorable attitudes towards vaccination are the underlying aim to protect the elderly and oneself (Rosman et al., 2021), as well as prioritizing the health of their fellow citizens in general (Coelho et al., 2021). Nevertheless, supporting the Covid-19 passport, may also ironically go against their ideals of protecting the more vulnerable, because it could result in involuntary discrimination against groups who traditionally are less likely to get vaccinated (Latkin et al., 2021a; Office for National Statistics, 2022) or people in countries who have not had a chance to get vaccinated (Ritchie et al., 2020; Schraer, 2021). Therefore, while a passport would discriminate against conservatives who freely choose not to get the vaccine, it could also discriminate, for instance, against Black people (Latkin et al., 2021a), as they are less likely to get vaccinated than conservatives.

As our results show, left-wingers perceive the passport as less discriminatory. We speculate that left-wingers’ positive attitudes towards vaccines (Kossowska et al., 2021) and the more significant benefits arising from its dissemination were weighted more heavily toward left-wingers’ positive attitudes towards the Covid-19 passport than any indirect discrimination. Another possibility is that the left-wingers were less aware of the consequences of the passport. While left-wingers generally tend to be more open toward new (scientific) information, they are motivated to reject scientific evidence inconsistent with their attitudes (Washburn & Skitka, 2018). Thus, having more favorable views towards vaccination might encourage them to ignore evidence of its negative implications. Future research is needed to unravel these hypotheses. For instance, researchers could directly ask to what extent left-wingers believe that people who belong to minority groups or from countries with poor access to Covid-19 vaccines are impacted by the passport and test whether this awareness moderates the prediction of attitudes towards the passport. Additionally, future research could manipulate whether a person is voluntarily or involuntarily unvaccinated. We predict that left-wingers are more likely to discriminate against voluntarily but not involuntarily unvaccinated people. While the former have freely chosen to contradict scientific evidence and might therefore be perceived as a threat to the progressive liberal agenda.

Beyond political orientation, we also found that certain values and moral foundations are important in predicting attitudes and perceived discrimination by the Covid-19 passport. Specifically, security, conformity, and self-direction were the only values that significantly explained the unique variance in these outcome measures. Security (e.g., health, social order) and conformity (e.g., self-discipline, obedience) positively predicted attitudes and negatively predicted perceived discrimination after controlling for political orientation, values, and moral foundations. In other words, those who highly endorse these values are more likely to perceive the passport as discriminatory and hold positive attitudes toward the passport. These values are part of the conservation dimension, and are characteristic of individuals motivated by the safety and stability of society and who restrain from violating social norms (Schwartz, 1992). In a pandemic context, one could expect that individuals holding these values would be more prone to care for their health and obey restrictions created to keep people secure, such as the ones introduced by national governments.

On the other hand, self-direction (e.g., freedom, independence) was negatively associated with attitudes and positively with perceived discrimination towards the passport after controlling for political orientation, values, and moral foundations. Such findings suggest that individuals who endorse independent thought and action (Schwartz, 1992) are more likely to oppose the passport and perceive it as discriminatory, likely because the passport restricts people’s freedoms.

Finally, only the care and loyalty foundations helped to explain the Covid-19 variables. High levels of the care foundation represent virtues of kindness, gentleness, and nurturance, whereas loyalty is linked to patriotism and self-sacrifice (Graham et al., 2011; Haidt & Graham, 2007). Care was positively associated with attitudes towards the passport and negatively with perceived discrimination of the passport, while the opposite pattern emerged for loyalty. In other words, individuals who endorse care are more likely to perceive the Covid-19 passport as a positive tool and not discriminatory. Such findings suggest that the greater good that the passport represents, of people being vaccinated and returning to their lives, outweighs any involuntary discrimination it may cause for people scoring high on the care foundation. On the other hand, individuals who score higher on loyalty tend to dislike the passport more. One potential explanation for such findings is that the passport might restrict them from being with the ingroups they are loyal to.

### Cross-cultural differences

First, we assessed whether there were statistical differences across countries in attitudes towards and perceived discrimination against the Covid-19 passport. Brazil was the only country that significantly differed from the others in both Covid variables: Participants living in Brazil reported more positive attitudes and lower perceived discrimination towards the passport. Our findings align with other research showing that Brazilians have a low level of vaccine hesitancy, with only 10.5% being concerned regarding its efficacy, fear of reaction, or any other reason (Moore et al., 2021). This might be a response to the detrimental impact Covid-19 had on Brazil. Indeed, the country was ranked the worst in managing Covid-19 (Lowy Institute, 2021). Further, other vaccines, such as polio, have been successfully distributed among Brazilians (Domingues et al., 2020), which may have helped shape a more positive attitude towards the Covid-19 vaccine and measures to enforce them, such as the use of the passport, and lower discrimination towards it. This might also explain the lower negative, albeit still significant, correlation between attitudes towards the passport and whether the passport discriminates: There were ceiling effects for attitudes, thus leaving less variance to be explained by perceived discrimination. Further, the Covid-19 passport was less discussed and used in Brazil, making it less relevant to people living in Brazil.

Notwithstanding, it is also essential to highlight why other countries presented less positive attitudes and a higher perception of discrimination regarding the passport. In the USA, for instance, this might have occurred due to the significant levels of vaccine distrust (Latkin et al., 2021b), and the consequent understanding that the use of Covid-19 passports would unfairly undermine peoples’ freedom to come and go. One potential takeaway from these findings is that future work and policy may focus on increasing public acceptance of the Covid-19 passport and other pandemic-related measures in countries such as the US and the UK, given that they revealed greater skepticism than countries such as Brazil.

### Implications and conclusion

Our findings have implications for policymakers who wish to elicit widespread societal acceptance of measures that keep the population safe and require solidarity and restriction among the public. The Covid-19 passport has been one such measure, but similar measures may be needed again in the event of future pandemics or other societal crises. Since attitudes have been found to significantly predict behavior (Armitage & Conner, 2001), our research indicates the factors underpinning individuals’ reluctance to follow a measure such as the passport. Thus, policymakers may benefit from using established measures to increase support, such as transparent communication (Enria et al., 2021) from trustworthy sources (Janssen et al., 2021), combined with tailored messages that directly aim at individuals with a more right-wing political orientation and people low in security values, for instance. Moreover, policymakers may wish to communicate more clearly that not all failure to comply with such a measure is under people’s control, for instance, in the case of younger age groups having no access to the vaccine or some groups objecting for religious reasons. Improving such communication may help avoid unintended discrimination and allow more social harmony during trying times.

Finally, our findings provide the first evidence of the associations between political orientation and attitudes toward the Covid-19 passport and whether people perceive it as a way to discriminate against unvaccinated people. Left-wingers, often caring about the social aspects of the community, have more positive attitudes towards the passport than right-wingers and perceive it as less discriminatory, perhaps because they, often incorrectly, perceive attaining the passport as under individuals’ control. Our findings provide novel insights into the conditions under which left-wingers support measures that can involuntarily discriminate against certain groups, such as underrepresented groups. For right-wingers, using the passport might be seen as standing in the way of traditions and customs. Beyond political orientation, we also found that specific human values (e.g., security) and moral foundations (e.g., care) are linked with attitudes towards the Covid-19 passport and perceived discrimination through its introduction in society. Policymakers may benefit from the awareness of other psychological factors predisposing individuals to greater reluctance to societal measures aimed at protecting the public. They may wish to tailor or adjust their communication to facilitate widespread societal acceptance of the measures.

## Supplementary information

Below is the link to the electronic supplementary material.

ESM 1 (DOCX 46.1 KB)

## Footnotes

1 Low internal consistencies are common when a broad construct is measured (Graham et al., 2011).
