# Supplementary material for: Do left-wingers discriminate? A cross-country study on the links between political orientation, values, moral foundations, and the Covid-19 passport
Source: Curr Psychol. 2023 Mar 21:1–12. Online ahead of print. doi: 10.1007/s12144-023-04554-9 (PMC10028313; doi:10.1007/s12144-023-04554-9)
Supplement: Supplementary file 1 — (DOCX 46.1 KB) [file 12144_2023_4554_MOESM1_ESM.docx]

| **Supplemental Table S1**  *Demographics per Country*. |  |  |  |  |
| --- | --- | --- | --- | --- |
| **Country** | **US** | **BR** | **UK** | **Others** |
| **N** | 199 | 233 | 105 | 137 |
| **Mage (SDage)** | 32.05 (12.18) | 31.62 (12.78) | 35.23 (13.05) | 31.11 (11.22) |
| **Gender** |  |  |  |  |
| *Man* | 65 | 92 | 43 | 62 |
| *Woman* | 116 | 131 | 60 | 72 |
| *Non-Binary* | 14 | 7 | 2 | 3 |
| *Prefer not to say* | 1 | - | 1 | 1 |
| *Other (Self-describe)* | 3 | - | - | 2 |
| **Political Orientation** |  |  |  |  |
| *Extreme Left* | 32 | 5 | 9 | 13 |
| *Left* | 60 | 88 | 24 | 44 |
| *Centre-Left* | 24 | 54 | 16 | 42 |
| *Centre* | 20 | 36 | 14 | 23 |
| *Centre-Right* | 16 | 18 | 14 | 6 |
| *Right* | 32 | 14 | 24 | 6 |
| *Extreme Right* | 14 | 3 | 4 | 3 |
| **Vaccination Status** |  |  |  |  |
| *Fully vaccinated* | 129 | 83 | 56 | 96 |
| *Only first dose (for vaccines that require two)* | 4 | 135 | 8 | 11 |
| *Not vaccinated yet, but I intend to get vaccinated soon.* | 2 | 7 | 1 | 10 |
| *Not vaccinated and I will not take the vaccine.* | 62 | 5 | 41 | 23 |
| **Do you know what is the Covid Passport?** |  |  |  |  |
| *Yes* | 175 | 179 | 103 | 136 |
| *No* | 24 | 54 | 3 | 4 |

## Multidimensional scaling

The common space plot is displayed below. We used Torgerson as initial configuration. The four higher order value types of Schwartz’s (1992) model conservation (conformity, tradition, security), self-transcendence (benevolence, universalism), openness (self-direction, stimulation, hedonism), and self-enhancement (achievement, power) clearly emerged. Some minor deviations occurred: Security is predicted to be closer to self-enhancement values and stimulation closer to self-direction than hedonism. But minor deviations within each higher-order value type are not uncommon and can be neglected (Bilsky et al., 2011; Schwartz & Sagiv, 1995).


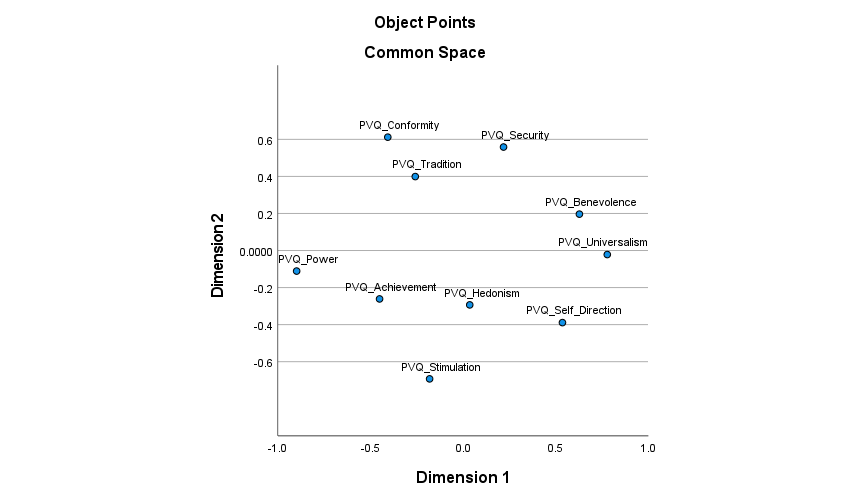


## References

Bilsky, W., Janik, M., & Schwartz, S. H. (2011). The structural organization of human values—Evidence from three rounds of the European Social Survey (ESS). *Journal of Cross-Cultural Psychology*, *42*(5), 759–776. https://doi.org/10.1177/0022022110362757

Schwartz, S. H. (1992). Universals in the content and structure of values: Theoretical advances and empirical tests in 20 countries. *Advances in Experimental Social Psychology*, *25*, 1–65. https://doi.org/10.1016/S0065-2601(08)60281-6

Schwartz, S. H., & Sagiv, L. (1995). Identifying culture-specifics in the content and structure of values. *Journal of Cross-Cultural Psychology*, *26*(1), 92–116. https://doi.org/10.1177/0022022195261007

| Model Coefficients - Pass_Discrimination | | | | | | | | | |
| --- | --- | --- | --- | --- | --- | --- | --- | --- | --- |
|  |  |  |  |  |  |  |  |  |  |
| **Predictor** | | **Estimate** | | **SE** | | **t** | | **p** | |
| Intercept ᵃ |  | 2.150 |  | 0.419 |  | 5.132 |  | < .001 |  |
| GROUPS_COUNTRY: |  |  |  |  |  |  |  |  |  |
| US – Brazil |  | 0.182 |  | 0.544 |  | 0.335 |  | 0.738 |  |
| UK – Brazil |  | 0.988 |  | 0.699 |  | 1.413 |  | 0.158 |  |
| Others – Brazil |  | -0.232 |  | 0.655 |  | -0.354 |  | 0.723 |  |
| Political_Orientation |  | 0.763 |  | 0.123 |  | 6.188 |  | < .001 |  |
| GROUPS_COUNTRY ✻ Political_Orientation: |  |  |  |  |  |  |  |  |  |
| (US – Brazil) ✻ Political_Orientation |  | 0.433 |  | 0.152 |  | 2.854 |  | 0.004 |  |
| (UK – Brazil) ✻ Political_Orientation |  | 0.331 |  | 0.181 |  | 1.833 |  | 0.067 |  |
| (Others – Brazil) ✻ Political_Orientation |  | 0.551 |  | 0.198 |  | 2.786 |  | 0.005 |  |
| ᵃ Represents reference level | | | | | | | | | |
|  | | | | | | | | | |

| Model Coefficients - Att_Towards_Passport | | | | | | | | | |
| --- | --- | --- | --- | --- | --- | --- | --- | --- | --- |
|  |  |  |  |  |  |  |  |  |  |
| **Predictor** | | **Estimate** | | **SE** | | **t** | | **p** | |
| Intercept ᵃ |  | 6.855 |  | 0.319 |  | 21.521 |  | < .001 |  |
| GROUPS_COUNTRY: |  |  |  |  |  |  |  |  |  |
| US – Brazil |  | 0.451 |  | 0.414 |  | 1.090 |  | 0.276 |  |
| UK – Brazil |  | -0.232 |  | 0.531 |  | -0.436 |  | 0.663 |  |
| Others – Brazil |  | 0.740 |  | 0.498 |  | 1.486 |  | 0.138 |  |
| Political_Orientation |  | -0.408 |  | 0.094 |  | -4.356 |  | < .001 |  |
| GROUPS_COUNTRY ✻ Political_Orientation: |  |  |  |  |  |  |  |  |  |
| (US – Brazil) ✻ Political_Orientation |  | -0.470 |  | 0.115 |  | -4.080 |  | < .001 |  |
| (UK – Brazil) ✻ Political_Orientation |  | -0.334 |  | 0.137 |  | -2.434 |  | 0.015 |  |
| (Others – Brazil) ✻ Political_Orientation |  | -0.576 |  | 0.150 |  | -3.831 |  | < .001 |  |
| ᵃ Represents reference level | | | | | | | | | |
|  | | | | | | | | | |
